# A Rare Case of Ileal Obstructive Trichobezoar in a Nine-Year-Old Child

**DOI:** 10.7759/cureus.109922

**Published:** 2026-05-30

**Authors:** Mohamed Taha Mellouki, Zineb Benmassaoud, Hind Cherrabi

**Affiliations:** 1 Department of Pediatric Surgery, Biotechnology and Medicine Laboratory, Souss Massa University Hospital Center, Faculty of Medicine and Pharmacy, Ibn Zohr University, Agadir, MAR

**Keywords:** child, ct scan, small bowel obstruction, trichobezoar, trichophagia

## Abstract

Trichobezoar is a rare gastrointestinal condition caused by the accumulation of ingested hair within the digestive tract, most commonly affecting children and adolescents with underlying behavioral disorders. Its diagnosis can be challenging because of nonspecific clinical manifestations and limited awareness of associated psychiatric conditions in pediatric patients. We report the case of a nine-year-old boy presenting with acute small bowel obstruction secondary to a terminal ileal trichobezoar, in whom the diagnosis was suggested by abdominal computed tomography and confirmed during surgery. The trichobezoar was successfully removed through enterotomy performed 10 cm proximal to the ileocecal valve. This case highlights the diagnostic value of preoperative imaging, the importance of complete intraoperative gastrointestinal exploration, and the need for psychiatric follow-up to prevent recurrence. It also emphasizes the unusual location of the trichobezoar and the diagnostic difficulties associated with this rare presentation.

## Introduction

Trichobezoar is a rare form of bezoar caused by the accumulation of ingested hair within the gastrointestinal tract. It mainly affects children and adolescent girls with underlying psychiatric or behavioral disorders, particularly trichotillomania and trichophagia. Because hair is resistant to digestion and peristalsis, it progressively accumulates within the digestive lumen, most commonly in the stomach, where it may form a compact mass. In some cases, the trichobezoar may extend beyond the pylorus into the small bowel, a condition referred to as Rapunzel syndrome [1,2].

The clinical manifestations of trichobezoar are often nonspecific and may include abdominal pain, vomiting, weight loss, anorexia, constipation, or signs of intestinal obstruction. As a result, diagnosis is frequently delayed, especially in pediatric patients in whom behavioral disorders may remain unrecognized [3-5]. Several imaging modalities can contribute to the diagnosis, including abdominal ultrasound and computed tomography (CT), while upper gastrointestinal endoscopy remains the gold standard for gastric trichobezoars [6-8]. Management depends on the size and location of the bezoar and may involve endoscopic or surgical treatment, particularly in complicated cases presenting with bowel obstruction or perforation [9].

This report aims to improve awareness of atypical presentations of trichobezoar in children and to discuss the diagnostic and therapeutic challenges associated with this rare condition. It also highlights the importance of appropriate preoperative evaluation, thorough intraoperative exploration of the gastrointestinal tract, and multidisciplinary postoperative management, including psychiatric follow-up.

## Case presentation

The patient was first seen in September 2024. A nine-year-old boy, with no significant past medical history except for untreated constipation, was admitted for persistent vomiting for one week. Initially food-related, the vomiting became bilious and was associated with cessation of stool and flatus for 48 hours. Clinical examination revealed diffuse abdominal distension without palpable masses, in a context of anorexia and a significant unintentional weight loss of 18 kg over three months.

An abdominal plain radiograph (Figure 1) showed features of small bowel obstruction, including a few air-fluid levels and absence of distal bowel gas. Abdominal ultrasound was limited due to bowel gas. Contrast-enhanced CT revealed an intraluminal mass in the terminal ileum, associated with proximal small bowel dilatation and moderate intraperitoneal fluid (Figure 2).

**Figure 1:**
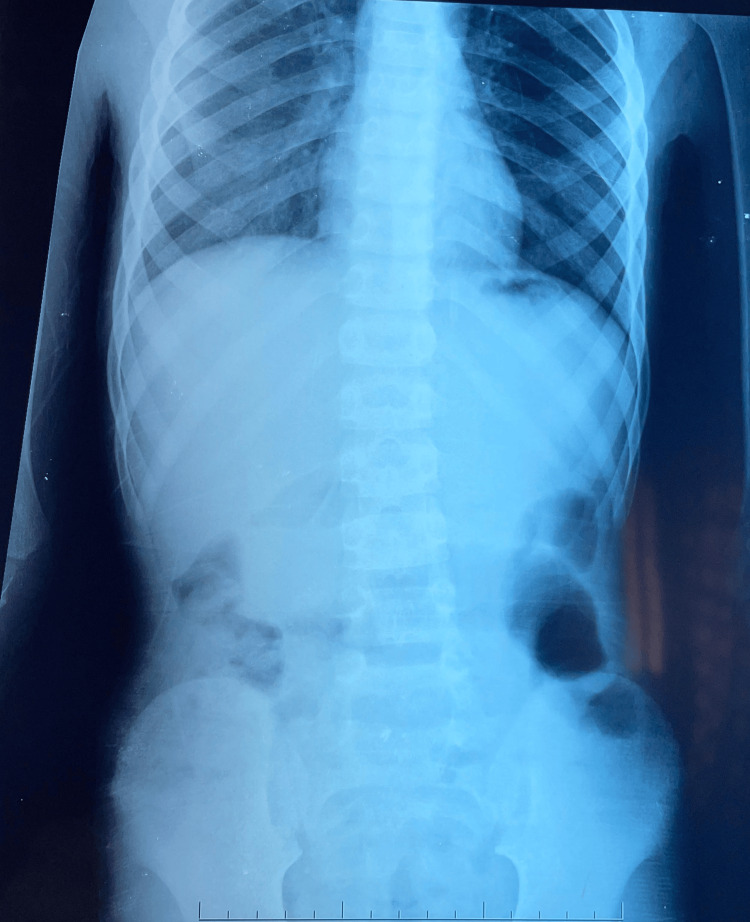
A few small bowel air-fluid levels with absence of gas in the remainder of the gastrointestinal tract

**Figure 2:**
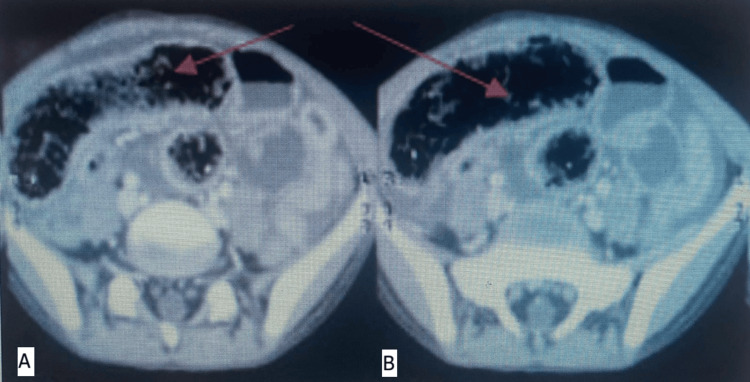
Abdominal computed tomography showing an oblong intraluminal mass within the bowel (arrow) (A) Axial computed tomography scan of the abdomen showing an intraluminal mass. (B) Computed tomography scan showing a mass occupying the intestinal lumen

Given the rapid progression to complete bowel obstruction and clinical deterioration, emergency surgery was indicated. A nasogastric tube was placed for decompression prior to surgery. A midline laparotomy revealed an intraluminal obstruction in the terminal ileum with upstream dilatation. The mass did not extend beyond the ileocecal valve. A transverse enterotomy was performed 10 cm proximal to the ileocecal valve, allowing complete extraction of a 35 cm trichobezoar (Figures 3-5).

**Figure 3:**
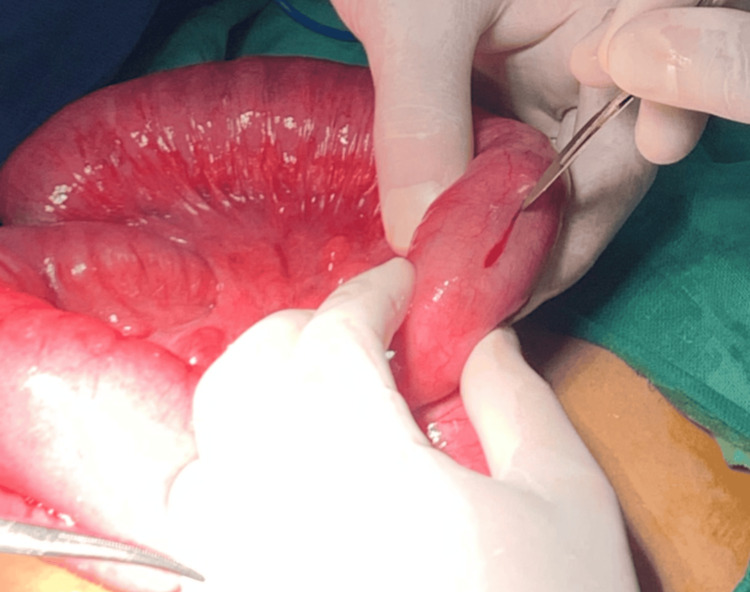
Intraoperative image showing the enterotomy site located at the most dilated segment of the bowel

**Figure 4:**
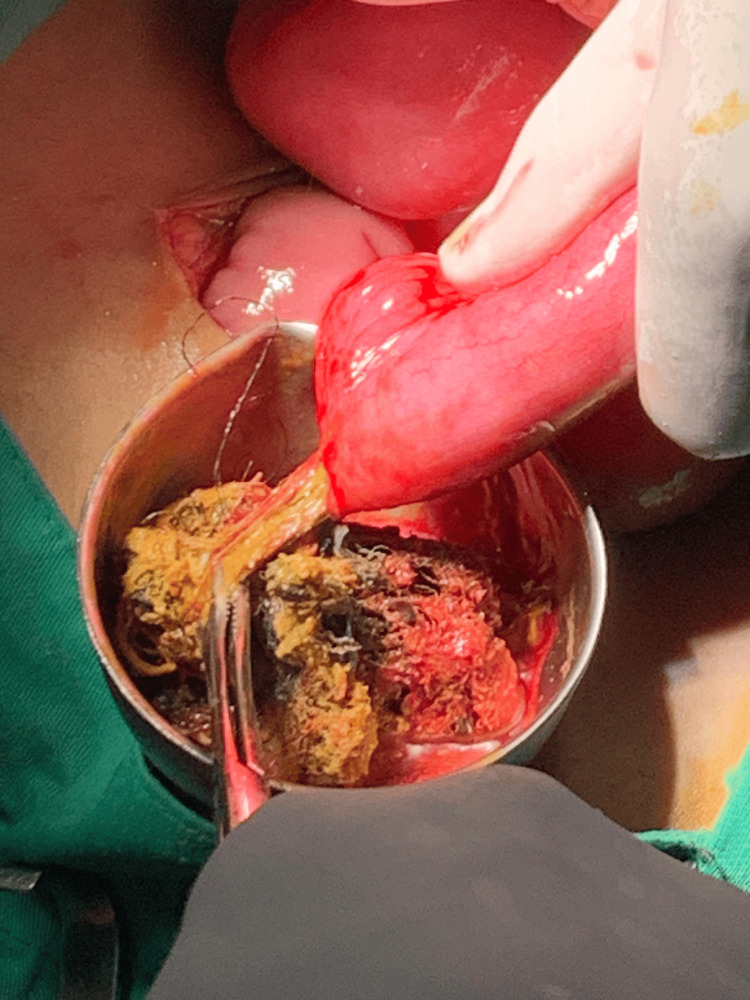
Initial removal of the trichobezoar during surgery

**Figure 5:**
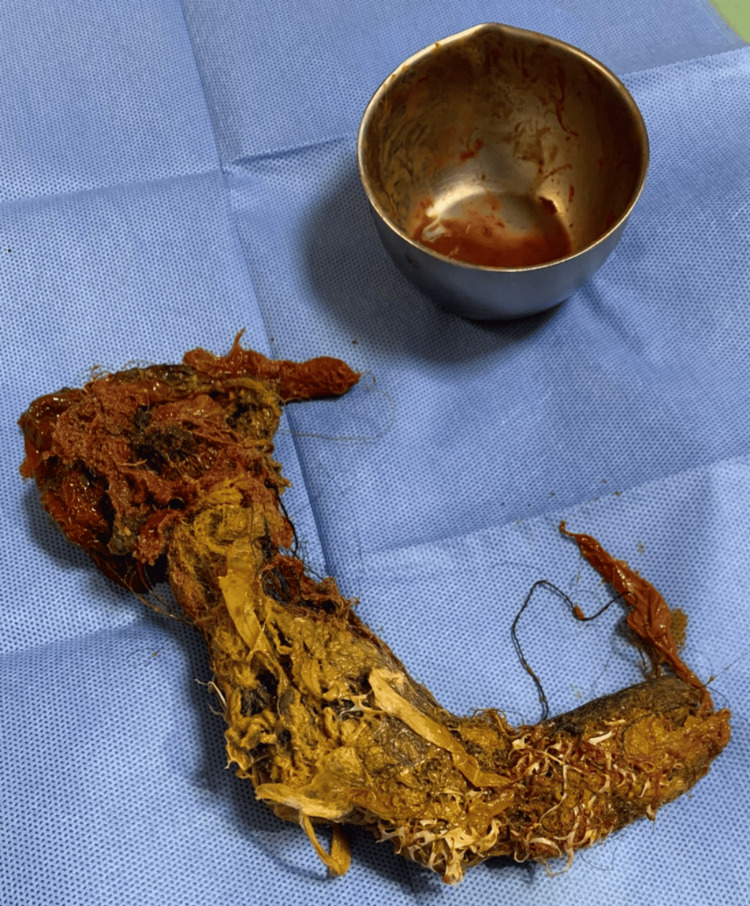
Entire trichobezoar after removal

Exploration of the entire gastrointestinal tract revealed no additional bezoars. The bowel wall was viable, allowing primary closure of the enterotomy (Figure 6). The postoperative course was uneventful. The patient was discharged on postoperative day 4 after resumption of bowel function and oral feeding. Psychiatric evaluation confirmed trichophagia, and appropriate management was initiated. At one-year follow-up, the patient remained in good general condition with satisfactory weight gain. He continues to receive psychiatric follow-up. Figure 7 summarizes the clinical timeline of the patient’s course from the initial presentation to long-term follow-up.

**Figure 6:**
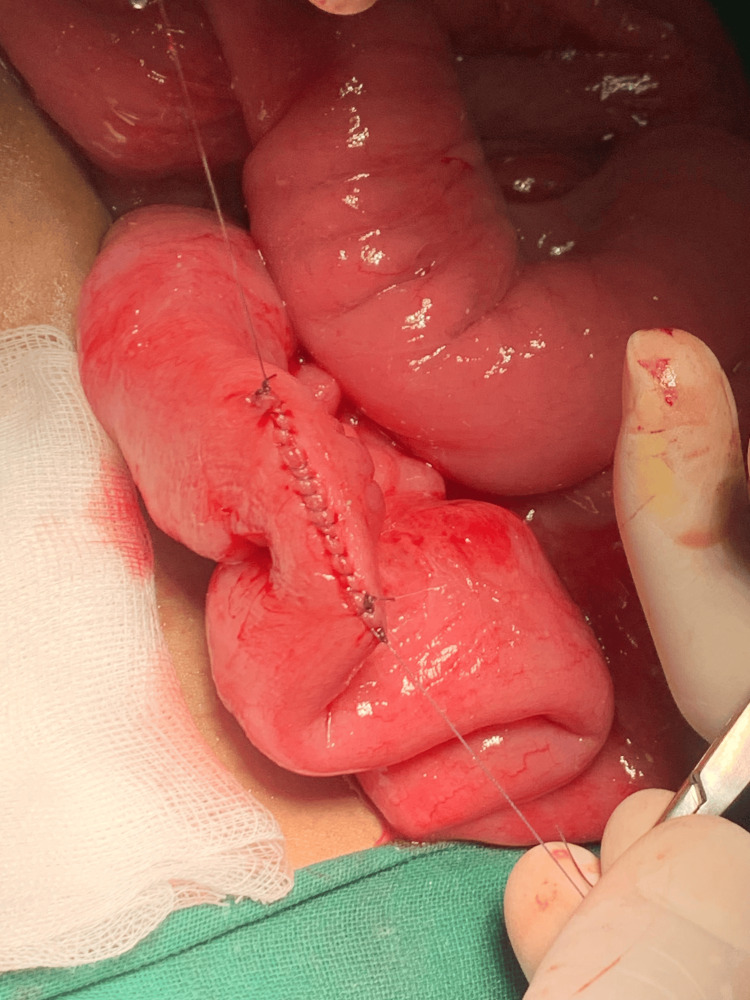
Intraoperative image of the enterotomy closure

**Figure 7:**
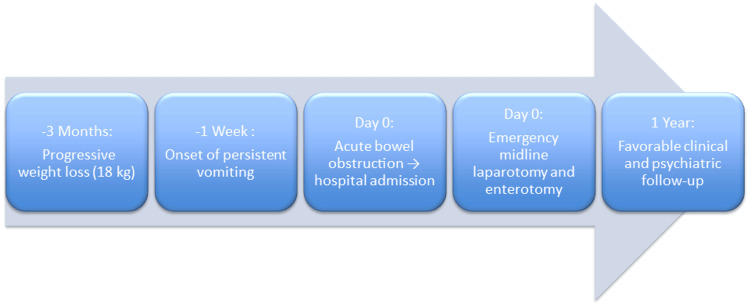
Clinical timeline of the patient’s course from initial symptoms to long-term follow-up Image credit: This is an original image created by the author Mohamed Taha Mellouki using Microsoft PowerPoint (Microsoft Corporation, Redmond, WA)

## Discussion

Trichobezoars remain rare entities, most commonly described in adolescent girls with psychiatric comorbidities, and typically originate in the stomach with possible extension into the small bowel in Rapunzel syndrome [1,2]. In contrast, isolated small bowel trichobezoars without gastric involvement are exceedingly rare, particularly in children, with only sporadic cases reported in the literature [3]. This makes our observation unusual in both location and clinical presentation.

In most reported cases, patients present with chronic and nonspecific symptoms, including abdominal pain, palpable abdominal mass, vomiting, anorexia, or weight loss, which may eventually progress to signs of gastric outlet obstruction. In distal forms, particularly jejunal or ileal trichobezoars, the clinical presentation is often acute and mimics other causes of small bowel obstruction, such as intussusception, adhesive obstruction, or foreign body ingestion, which contributes to diagnostic difficulty and delay [4].

Imaging, especially CT, plays a crucial role in the diagnostic workup by demonstrating a well-defined intraluminal mass with heterogeneous density and trapped air. In reported series, CT is highly sensitive for gastric trichobezoars and also contributes to the diagnosis of distal forms by identifying features suggestive of intraluminal obstruction, although precise etiological characterization may remain challenging. In our case, CT strongly suggested an intraluminal obstructive process of organic nature, allowing early surgical management [5,6].

Upper gastrointestinal endoscopy remains the gold standard for gastric trichobezoars, allowing both diagnosis and, in selected cases, therapeutic removal. However, its utility is limited for distal or large bezoars, for which surgical management is required [7].

Surgical removal remains the definitive treatment for complicated or distally located trichobezoars. Enterotomy allows complete extraction and should always be accompanied by systematic exploration of the entire gastrointestinal tract to exclude synchronous lesions [8,9].

The mechanisms underlying isolated distal localization remain unclear. Several hypotheses have been proposed, including the passage of small hair fragments through the stomach without retention, transient alterations in gastric motility, or fragmentation and distal migration of an initially gastric bezoar that may have gone unnoticed. These mechanisms may explain the absence of gastric involvement in our case, in contrast to the more typical Rapunzel syndrome.

Recurrence is strongly associated with persistent underlying psychiatric disorders, particularly trichotillomania and trichophagia, as consistently reported in the literature [10,11]. This highlights the importance of long-term psychiatric follow-up and multidisciplinary care.

Compared with previously reported cases, our observation is distinguished by its isolated ileal localization, acute obstructive presentation, and the diagnostic contribution of CT in guiding early surgical management. These features emphasize the importance of considering trichobezoar in the differential diagnosis of pediatric small bowel obstruction, even in the absence of gastric involvement, and underline the need for a multidisciplinary approach.

## Conclusions

Trichobezoar is a rare entity that may present with nonspecific and misleading clinical features, often resulting in delayed diagnosis. It can lead to significant complications, including gastrointestinal obstruction, particularly in atypical or distal localizations. Diagnosis relies on imaging and endoscopic evaluation, while definitive management is most often surgical in complicated cases.
